# Mental resources, mental health and sociodemography: a cluster analysis based on a representative population survey in a large German city

**DOI:** 10.1186/s12889-023-16714-4

**Published:** 2023-09-20

**Authors:** Kristine Khachatryan, Daniëlle Otten, Manfred E. Beutel, Sven Speerforck, Steffi G. Riedel-Heller, Christine Ulke, Elmar Brähler

**Affiliations:** 1 Department of Psychosomatic Medicine and Psychotherapy, University Medical Center, Johannes Gutenberg-University, Untere Zahlbacher Str. 8, 55131 Mainz, Germany; 2 Department of Psychiatry and Psychotherapy, Leipzig University Medical Center, Leipzig, Germany; 3 Institute of Social Medicine, Occupational Health and Public Health, Faculty of Medicine, University of Leipzig, Philipp-Rosenthal-Str. 55, Haus W, 04103 Leipzig, Germany

**Keywords:** Mental health, Mental resources, Optimism, Social support, Cluster analyses, East Germany

## Abstract

**Background:**

Mental resources such as optimism and social support are important to face different stressors. The aim of this study is to identify groups in the population that are similar in terms of their mental resources.

**Methods:**

For this purpose, a randomly selected general population community sample was used, representative for the city of Leipzig, Germany. In a two-stage process, three clusters were identified using hierarchical cluster analysis and the K-means method and then tested with a multinomial logistic regression analysis for differences in sociodemographic characteristics.

**Results:**

Three clusters were identified which vary in their extent of social support and optimism. In distinguishing between those with higher and lower (medium or poor) mental resources, male gender, unemployment, being born abroad and low household income are risk factors for having fewer mental resources. Internal migrants from West Germany and persons with children at home have a higher chance of being in the type with good mental resources. The groups with medium and lower mental resources differ significantly only by variables living with a partner and employment.

**Conclusion:**

Our results indicate that good mental resources are associated with good mental health. Special mental health care programs, focusing in particular on the needs of vulnerable groups with poor mental resources within a society, should be implemented.

## Background

In a rapidly changing and stressful world, the challenge to cope with different stressors is ever more pressing. In order to deal with such stressors, people need to acquire resources. These resources do not (only) concern material resources, but also psychological and social resources. Social networks, job, income and level of education can both fight stressors as resources [1, 2] and in turn be the cause of some stressors [3]. Altogether, material, psychological and social resources could contribute to overcome daily stress.

### Optimism and social support as protective factors for mental health

Optimism is a psychological resource and reflects “the extent to which people hold generalized favorable expectancies for their future” [4]. Optimism goes hand in hand with good physical and mental health. An optimistic attitude contributes to take steps to protect one’s own health [4]. For example, in patients with Parkinson’s disease, it has been empirically confirmed that positive illness perceptions predicted better well-being [5]. In older adults, the relationship between illness burden and anxiety symptoms weakens with high optimism and strengthens with high pessimism [6]. In patients with stages III-IV cancer undergoing active chemotherapy, greater optimism and self-efficacy were associated with less negative illness appraisal, less avoidant coping, and decreased mood disturbance [7]. According to Carver et al. [4] “the energetic, task-focused approach” of optimists to their goals also seems to be beneficial in socio-economic terms. A study on former law students indicates that dispositional optimism results in long-term resource growth (high salary). In addition, the growth of the social network increases the optimism [8]. Robb et al. [9] found a positive association between socioeconomic status (SES) and dispositional optimism. Looking at the subscales for optimism and pessimism separately, the results show that association with SES is strong for pessimism and minimal for optimism.

Social support is a social resource associated with quality of life. It is particularly evident in adaptation and recovery from physical illness and for the prognosis and status of chronic diseases [10–13]. Especially for depressed patients, social support plays an important role in adaptation and coping with an illness [14, 15]. There is also empirical evidence that the lack of social support contributes to new onset or recurrence of depressive symptoms [16–18]. Thus, social support and mental health are highly related [19–21]. Social support is not equally distributed among the population. In a large-scale German health study conducted between 2014 and 2015, older people, low-educated and economically inactive women and men reported comparatively low levels of social support [22].

Although psychological and social resources have a significant impact on health, their effect varies depending on the socioeconomic status of the person. In a study by Schöllgen et al. [23] psychological resources (self-esteem, control beliefs, optimism) were stronger predictors of functional and subjective health among people with low compared to higher levels of education. Social resources (perceived emotional and informational support, network size) were often associated with better functional and subjective health, mainly in people with a lower income. Results also showed that social resources were particularly important for financially disadvantaged older people [20].

### Specifics of East Germany in terms of mental health of the population

Because of the division of Germany after World War II into the German Democratic Republic (GDR) and the Federal Republic of Germany (FRG), a large part of the population of today’s Germany grew up under different socio-economic and political conditions, which left their mark on the population’s mental health [24–28]. East German states faced a lot of uncertainties after German reunification in 1990. For many East Germans, it was difficult to secure their working and living conditions, especially in the first few years after reunification [29]. Unemployment, which was practically non-existent in the former German GDR, reached significant levels after reunification [30]. Massive emigration of qualified young people from East to West Germany particularly affected structurally weaker regions in East Germany [31]. Empirical studies indicate a gradually significant decrease in mental burden in the East German population since German reunification [28]. Many of the differences between East and West Germany in mental health of the population that existed at the time of reunification have smoothed out over time [24, 32]. At the same time, there are still differences between East and West in some aspects of mental health. For example, East Germans show a lower general life satisfaction. Wealth difference between East and West Germans has increased over time and negatively impacted the general life satisfaction of East Germans [33]. Before the onset of the COVID-19 pandemic, self-reported loneliness was higher in eastern German states than western states [34]. Cordes et al. [10] found that lower scores in perceived social support are more common among East Germans than West Germans. At the same time, there were also protective factors in the social system of the former GDR that favored mental health. For example, equal distribution of roles at work [35] and social support in childcare [36] enable motherhood in full-time employment.

### Grouping people based on mental ressources

People with weak or strong mental resources are identified by their assessed characteristics based on empirically tested cut-off thresholds. The disadvantage here is that further variations of the sample beyond the cut-off value are no longer taken into account. If people are grouped on the basis of only one characteristic, the relationships with other characteristics are not taken into account. Cluster analyses based on a multivariate approach offer the possibility to classify people according to their similarity in several characteristics. Their aim is to combine people into groups or clusters that are as similar as possible within the group and as dissimilar as possible between the groups with regard to the characteristics studied [37]. In this way, people with similar profiles can be identified, in which correlations between different characteristics are also taken into account. An empirical typology that takes both social and psychological resources into account, can make statements about the population’s disposal of mental resources. According to our literature research, there are hardly any empirical typologies with regard to mental resources. In the present study, we would like to address this knowledge gap.

The analysis is based on a German general population community sample, representative for a German city of Leipzig. Leipzig is a large city in the eastern German state of Saxony. The aim of this study is to apply cluster analyses to identify groups in the population with different mental resources and to describe them according to sociodemographic and psychological characteristics.

## Method

### Measurements

Cluster analyses were carried out using the measurement items of the social support (ESSI] and optimism (LOT-R) constructs. These instruments are briefly described below. The specified cut-off values were taken into account when describing the cluster with psychological characteristics.

#### Social support: German version of the ENRICHD Social Support Instrument (ESSI, [38, 39])

Perceived availability of people with important social functions was measured by ESSI. There were five items used to measure aspects of perceived social support on a scale ranging from 1 “never” to 5 “always”. Lack of social support was measured with a scale value ≤ 18 and at least 2 items ≤ 3 [10].

#### Dispositional optimism

Dispositional optimism was measured with the *Life Orientation Test-Revised* (LOT-R). The LOT-R ( [40], for the test of psychometric properties and population-based norms see [41, 42] consists of 10 items, three of which measure optimism and another three pessimism. The remaining four items are filter items that were not collected in the LIFE survey. All items were measured on a 5-point Likert scale ranging from 1 “strongly disagree” to 5 “strongly agree”. The two sub-dimensions optimism and pessimism were calculated for later description of the clusters by summing up the corresponding items and ranked as total scores from 3 to 15.

The clusters were described by the following psychological characteristics:

#### Depression (Center for Epidemiological Studies-Depression CES-D)

Mental distress of respondents was assessed using the Instruments Center for Epidemiological Studies-Depression (CES-D, [43]). The CES-D is a 20-item measure that asks how often certain symptoms associated with depression have been experienced in the past week. Response options ranged from 0 “rarely or none of the time (less than 1 day)” to 3 “most of the time (5 to 7 days)” for each item. Scores ranged from 0 to 60, with high scores indicating greater depressive symptoms. Sum scores of 16 and higher identify a high risk of clinical depression ( [44], for prevalence rates of depressive symptoms in the adult population of the city of Leipzig see [45]).

#### Somatic Symptoms (Patient Health Questionnaire-15, PHQ-15, [46, 47], for normative data see [48, 49])

Severity of somatic symptoms was measured with the PHQ-15. The questionnaire consists of 13 items on somatoform disorders and two items on depressive disorders. The item “menstrual pain or other menstrual problems” was not included in the LIFE survey. The evaluation of the somatic symptoms was thus carried out in this evaluation using 12 items. Questions about somatoform symptoms were collected on a scale ranging from 0 “not affected” to 2 “severely affected”. Participants were asked to indicate how often they were bothered by the respective symptom over the course of the last 4 weeks. Questions about depressive symptoms were measured on a scale ranging from 0 “not affected at all” to 3 “affected almost every day”. Respondents were asked about the frequency of symptoms over the past 2 weeks. For the purpose of creating an overall scale for PHQ symptoms, categories 2 “affected more than half of the days” and 3 “affected almost every day” for these two items were combined and thus a scale similar to the items on somataform symptoms was obtained. Following the scoring of the PHQ-15, we added scores in the range of 0–28. Cut-off points for low, medium, and high somatic symptom severity represented by PHQ-15 scores of 5, 10, 15, respectively [32].

#### Anxiety (Generalized Anxiety Disorder-7; GAD-7)

The GAD-7 ( [50], for psychometric evaluation see [51, 52] is a one-dimensional instrument designed to detect symptoms of generalized anxiety disorder as defined in the DSM-IV [53]. The item scores ranged from 0 ”not at all” to 3 “nearly every day”, resulting in a sum score range from 0 to 21. Clinically relevant symptom burden was defined as a sum score ≥ 10. In this evaluation we have a total score of 0–4 as *minimal anxiety*, score 5–9 as *mild anxiety*, score 10–14 as *moderate anxiety*; and finally score greater than 15 is recoded as *severe anxiety* in order to also be able to consider milder forms of anxiety.

### Socio-demographic characteristics

In the analysis, the following characteristics were used to describe the clusters: *gender* (male; female), *age group* (< 26 years; 26–35 years; 36–45 years; 46–55 years; 56–65 years; >66 years), *family status* (married, living together; married, living separately; single; divorced; widowed), *children in the household* (yes; no), *number of persons in the household* (1 person; 2 persons; 3 and more persons), *occupational status* (working full time; working part-time; unemployed; retired; other), *living with a partner* (yes; no), *net household income* (below average (median): < 2750 Euro); average or above average: ≥ 2750 Euro).

The *net equivalent income in the city district* was also included to describe the clusters, as there is empirical evidence that people from economically disadvantaged neighbourhoods report poorer mental health [54]. Information from the Social Report Leipzig from 2012 was included in this respect. The calculation of the net equivalent income in Sozialreport Leipzig was based on the new OECD scale [55], according to which the head of household receives a weight of 1.0 and each additional adult member a weight of 0.5. Children and young people under the age of 14 were included in the calculation with a weight of 0.3 [56]. This indicator was used in the Leipzig Social Report to identify districts with high and low equivalent net incomes, with the highest income district earning about twice as much as the lowest income district [56]. This information was used in the present study for the purpose of grouping the districts in the dataset accordingly. Districts of Leipzig “Zentrum-West”, “Schleußig”, “Engelsdorf”, “Heiterblick”, and “Plaußig-Portitz” were coded as high net equivalent income neighborhoods, and “Volksmarsdorf”, “Grünau-Nord”, and “Neustadt-Neuschoenefeld” were coded as low net equivalent income neighborhoods. The remaining Leipzig districts were coded as middle-income districts.

Furthermore, the *origin* of the study participants was included in the description of clusters, as there is empirical evidence of poorer mental health among immigrants [57–59]. In the case of persons born in Germany, the place of birth was used to check whether they were born in the former Federal Republic of Germany (FRG) or in the former German Democratic Republic (GDR). In addition, 1st and 2nd generation migrants were identified based on the country of birth of the study participants and their parents. Respondents who indicated a country other than Germany as their country of birth were coded as first-generation migrants. Respondents who reported being born in Germany, but whose mother or father were born abroad, were coded as second-generation migrants.

### Sample

Analysis was based on the LIFE-Adult-Study of the Leipzig Center for Civilization Diseases (LIFE) which was approved by the ethics committee of the University of Leipzig. It was an age- and gender-stratified random sample of people living in Leipzig, in the age group of 40 to 79 years. Leipzig is a city with about 550,000 inhabitants (at the time of the survey) in the east of Germany. The baseline examination was conducted from August 2011 to November 2014. The age group 18 to 39 year olds participated in the study underrepresented. Informed consent was obtained from all participants. Details on the sampling procedure are outlined in Loeffler et al. [60]. The response rate of the study was 33% (compared with similar studies [61–63], this level of participation would be considered satisfactory), with the resulting sample equal to 10,000 study participants.

In this study, we included only participants who did not have missing values in the items underlying the cluster formation. This lead to a sample size of *N* = 9,701 with a mean age of 57 year (SD = 12.43), including 52% women (n = 5,044). The distribution of gender was representative for the population in Leipzig in 2012 [64]. About 63% of the sample were married (*n* = 6,063), 18% were single (*n* = 1,746), 14% divorced (*n* = 1,329) and 6% widowed (*n* = 563). Of those who were not married 37% lived with a partner (*n* = 1,353). Around 45% of those surveyed worked full-time (*n* = 4,330), 12% part-time (*n* = 1,209), around 6% were unemployed (*n* = 554), 36% were retired (*n* = 3,441) and the remaining 2% were students, houshusbands or housewives (*n* = 167).

### Statistical analyses

The data were analyzed with the program IBM SPSS (version 27, [65]). Clusters were formed using the measurement items of the constructs social support (ESSI) and dispositional optimism (LOT-R). Social support and dispositional optimism thus were not considered as combined scales in cluster analyses. The cluster analyses were carried out directly on the basis of individual measurement indicators of these two constructs (a total of 11 items). The items were initially z-standardized (i.e., transformed into a distribution with mean 0 and standard deviation 1) to bring values ​​from different scales into a uniform format.

The classification was performed using two methods: hierarchical cluster analysis with Ward linkage and cluster analysis according to the K-means method [66]. A fundamental disadvantage of hierarchical cluster analysis is that clusters formed first are particularly homogeneous, which is at the expense of the heterogeneity of clusters formed later. In contrast, the K-Means method forms groups with a “medium” homogeneity [67]. At the same time, K-means-clustering requires preliminary information about the groups, such as the number of clusters. Therefore, both methods were combined in this study. In a first step, a hierarchical cluster analysis with allocation according to the Ward method was carried out. Based on the dendrogram and evaluation of the increase in heterogeneity in the groups, a decision was made in favor of a 3-cluster solution. Content plausibility of the clusters was also taken into account. This was followed by a cluster analysis using the K-Means method, in which the mean values ​​of the groups formed by hierarchical cluster analysis were used as starting values. The formed clusters were then described with sociodemographic characteristics as well as with constructs of mental burden and resources. Differences in psychological characteristics between the clusters were tested using non-parametric tests since the data used were not normally distributed [68]. The Kruskal-Wallis test (H-test) was first used to check whether there was a statisticaly significant difference between the clusters with regard to psychological characteristics. Then, the Mann-Whitney U test was used to make pairwise cluster comparisons of the characteristics for which the Kruskal-Wallis test was significant. Differences between the clusters with regard to sociodemographic characteristics were checked using the Pearson-Chi-square test. The statistical significance level was set at 5%. Subsequently, a multinomial logistic regression analysis was carried out to highlight features that are related to cluster affiliation.

## Results

### Clusters with different extent of mental resources

Table 1 provides information on the distribution of the items on which cluster analyses were based.

**Table 1:** Distribution of items for cluster analyses, *n* = 9,701

	Mean	Standard deviation	Skewness	Kurtosis
***LOT-R***^a^				
In uncertain times, I usually expect the best.	3.74	1.04	-0.50	-0.30
If something can go wrong for me, it will.	2.60	1.03	0.37	-0.48
I am always optimistic about my future.	4.02	1.0	-0.81	0.12
I hardly ever expect things to go my way.	2.43	0.96	0.44	-0.32
I rarely count on good things happening to me.	2.46	1.14	0.38	-0.76
Overall, I expect more good things to happen to me than bad.	4.03	1.07	-1.06	0.54
***ESSI***^b^				
Is there someone available to whom you can count on to listen to you when you need to talk?	4.52	0.77	-1.95	4.16
Is there someone available to you to give you good advice about a problem?	4.40	0.82	-1.55	2.49
Is someone available for you to show you love and affection?	4.39	0.93	-1.71	2.55
Can you count on anyone to provide you with emotional support (talking over problems or helping you make a difficult decision)?	4.40	0.86	-1.63	2.59
Do you have as much contact as you would like with someone you feel close to, someone in whom you can trust and confide in?	4.37	0.89	-1.59	2.29

^a^Measuring scale: 1 “not true at all”, 2 “hardly true”, 3 “partly true”, 4 “true”, 5 “very true”

^b^Measuring scale: 1 “never”, 2 “seldom”, 3 “sometimes”, 4 “mostly”, 5 “always”

Using two methods of clustering – hierarchical cluster analysis and the K-means method – three clusters were identified which vary in their extent of social support and optimism. Mean values of the z-standardized items of the clustering can be found in Table 2.

**Table 2:** Mean values of z-standardized items by cluster membership, *n* = 9,710

	Cluster 1		Cluster 2		Cluster 3	
	Mean	Standard deviation	Mean	Standard deviation	Mean	Standard deviation
Is there someone available to whom you can count on to listen to you when you need to talk?	0.41	0.49	0.09	0.66	-1.85	1.23
Is there someone available to you to give you good advice about a problem?	0.42	0.57	0.06	0.72	-1.80	1.07
Is someone available for you to show you love and affection?	0.40	0.54	0.07	0.78	-1.76	1.11
Can you count on anyone to provide you with emotional support (talking over problems or helping you make a difficult decision)?	0.44	0.52	0.08	0.67	-1.94	1.00
Do you have as much contact as you would like with someone you feel close to, someone in whom you can trust and confide in?	0.39	0.58	0.08	0.75	-1.78	1.11
In uncertain times, I usually expect the best.	0.55	0.75	-0.52	0.91	-0.41	0.96
If something can go wrong for me, it will.	-0.30	0.94	0.26	0.97	0.33	0.98
I am always optimistic about my future.	0.59	0.61	-0.50	0.95	-0.63	1.06
I hardly ever expect things to go my way.	-0.46	0.84	0.42	0.93	0.42	0.98
I rarely count on good things happening to me.	-0.44	0.93	0.40	0.88	0.41	0.90
Overall, I expect more good things to happen to me than bad.	0.52	0.71	-0.49	0.99	-0.41	0.97

*Cluster 1* High-resource group, *Cluster 2* Group with medium mental resources, *Cluster 3* Low-resource group

Clusters with good, medium and poor mental resources were identified. Clusters with medium and poor mental resources vary in their level of social support. In terms of optimism, these two clusters do not differ statistically (see Tables 3, 4 and 5).

**Table 3:** Psychological resources and mental burden of clusters; mean (SD), *n* = 9,710

	Cluster 1 (*n* = 4689)	Cluster 2 (*n* = 3837)	Cluster 3 (*n* = 1175)	Total (*N* = 9701)	Kruskal-Wallis-H (df), *p*
Active characteristics (cluster analyses were carried out with their indicators)					
ESSI	23.82 (1.69)	22.38 (2.20)	14.20 (3.15)	22.09 (3.68)	4007.31 (2) *p* < 0.01
LOT-R Optimism	13.47 (1.42)	10.18 (2.02)	10.26 (2.45)	11.78 (2.45)	4721.29 (2) *p* < 0.01
LOT-R Pessimism	6.24 (1.96)	8.63 (2.08)	8.72 (2.30)	7.49 (2.38)	2614.26 (2) *p* < 0.01
Passive characteristics (used only to describe the clusters)					
GAD7	2.47 (2.45)	4.14 (3.55)	5.72 (4.16)	3.52 (3.35)	958.29 (2) *p* < 0.01
CES-D	7.87 (4.99)	12.38 (6.71)	16.48 (8.63)	10.63 (6.89)	1702.37 (2) *p* < 0.01
PHQ15	4.40 (3.26)	6.07 (3.99)	7.32 (4.42)	5.38 (3.85)	602.53 (2) *p* < 0.01
SWLS	28.87 (3.97)	25.15 (5.36)	21.26 (6.37)	26.48 (5.54)	2005.2 (2) *p* < 0.01

Characteristics are referred to as active here if they or their indicators serve as the basis for cluster formation

Characteristics that were only included to describe the finished clusters are referred to as passive

*Cluster 1* High-resource group, *Cluster 2 *Group with medium mental resources, *Cluster 3* Low-resource group

**Table 4:** Non-parametric tests to compare the mean values of psychological constructs of the clusters 1 and 2, *n* = 8,526

	ESSI	CES-D	PHQ15	SWLS	GAD7	LOT-R Optimism	LOT-R Pessimism
Mann-Whitney-U-Test	5,588,475	4,204,154	5221606.5	501,328.,5	6,040,790	1,632,098	3622943.5
Z	-31.44	-33.09	-19.31	-34.00	-22.94	-65.76	-47.90
Asymp. Sig. (2-sided)	< 0.01	< 0.01	< 0.01	< 0.01	< 0.01	< 0.01	< 0.01

*Cluster 1* High-resource group, *Cluster 2* Group with medium mental resources

**Table 5:** Non-parametric tests to compare the mean values of psychological constructs of the clusters 2 and 3, *n* = 5,012

	ESSI	CES-D	PHQ15	SWLS	GAD7	LOT-R Optimism	LOT-R Pessimism
Mann-Whitney-U-Test	7007	1242927.5	1338333.5	1,406,049	1,625,389	2,217,879	2,190,696
Z	-52.17	-14.46	-8.10	-18.55	-11.96	-0.85	-1.48
Asymp. Sig. (2-sided)	< 0.01	< 0.01	< 0.01	< 0.01	< 0.01	0.397	0.139

*Cluster 2* Group with medium mental resources, *Cluster 3* Low-resource group

**Table 6:** Sociodemographic composition of clusters, *n* = 9,701

	Cluster 1 (*n* = 4,689)	Cluster 2 (*n* = 3,837)	Cluster 3 (*n* = 1,175)	Total (*N* = 9,701)	Pearson-Chi-Quadrat (df), *p*
***Gender***					18.87 (2), *p* < 0.01
Male	45.9%	49.0%	52.3%	**47.9%**	
Female	54.1%	51.0%	47.7%	**52.1%**	
***Age group***					83.09 (10), *p* < 0.01
< 26 years	1.4%	1.7%	0.5%	**1.4%**	
26–35 years	3.2%	2.5%	2.2%	**2.8%**	
36–45 years	18.5%	14.1%	15.7%	**16.4%**	
46–55 years	26.9%	24.4%	27.9%	**26.0%**	
56–65 years	23.2%	24.3%	27.1%	**24.1%**	
≥ 66 years	26.8%	33.0%	26.6%	**29.2%**	
***Family status***					321.78 (8), *p* < 0.01
Married, living together	65.1%	61.0%	37.4%	**60.1%**	
Married, living separately	2.2%	2.2%	4.3%	**2.4%**	
Single	16.4%	17.0%	27.6%	**18.0%**	
Divorced	11.6%	13.2%	23.4%	**13.7%**	
Widowed	4.8%	6.6%	7.4%	**5.8%**	
Living with partner (if not married)	47.8%	37.3%	13.5%	**37.2%**	256.70 (2), *p* < 0.01
***Occupational status***					310.65 (12), *p* < 0.01
Working full time (with 35 h and more/ week)	50.5%	39.4%	38.3%	**44.6%**	
working part-time with 15 to 34 h/week	9.9%	9.5%	9.0%	**9.6%**	
part-time/hourly employed with less than 14 h/week	2.5%	3.3%	2.5%	**2.8%**	
Retired	32.1%	39.8%	34.7%	**35.5%**	
Unemployed	3.3%	6.1%	14.2%	**5.7%**	
Houseman/housewife/maternity leave/parental leave	0.6%	0.9%	0.5%	**0.7%**	
Study	1.1%	1.0%	0.8%	**1.0%**	
***Persons in household***					650.32 (4), *p* < 0.01
1 person	15.4%	22.3%	48.8%	**22.2%**	
2 persons	59.7%	60.1%	36.4%	**57.0%**	
3 or more persons	25.0%	17.6%	14.8%	**20.8%**	
**Children in household (yes)**	19.0%	13.5%	19.1%	**16.9%**	38.92 (2), *p* < 0.01
***Household income***					242.16 (2), *p* < 0.01
< 2750 Euro /Month	36.4%	54.1%	58.2%	**45.2%**	
≥ 2750 Euro /Month	63.6%	45.9%	41.8%	**54.8%**	
***Origin***					19.75 (6), *p* < 0.01
FRG	4.8%	3.4%	3.5%	**4.1%**	
GDR	77.3%	77.7%	76.5%	**77.3%**	
1st Generation migrants	1.6%	2.9%	2.8%	**2.3%**	
2nd Generation migrants	16.3%	16.0%	17.1%	**16.3%**	
***Net equivalent income in district***					11.25 (4), *p* < 0.05
Low	4.1%	5.1%	6.0%	**4.7%**	
Medium	90.7%	88.9%	88.5%	**89.7%**	
High	5.2%	6.0%	5.5%	**5.6%**	

*Cluster 2* Group with medium mental resources, *Cluster 3* Low-resource group

In the following, individual clusters are described in detail, taking into account not only the variables underlying the clustering, but also sociodemographic characteristics and indicators of mental burden (see Tables 3 and 6). In addition to the values for individual clusters, values are also given for the total sample in order to detect above- or below-average distributed characteristics in the clusters. Only statistically significant differences between the groups are described.

#### Cluster 1: High-resource group (48.3%)

Persons belonging to this cluster report an above-average amount of social support and optimism with limited pessimism at the same time. Further, this cluster reports a high general life satisfaction. Compared to the other clusters, somatic symptoms, depression, and anxiety are rare within this cluster (see Table 3).

With regard to sociodemographic features (see Table 6), women and young age groups are over-represented in this cluster. Married people and people living with a partner are also more common in this group. Accordingly, single households are less common in this cluster, whereas households with 3 or more people are more widely represented. Internal migrants from West Germany are more common among this group than other groups. First-generation migrants are relatively rare here. With regard to socioeconomic features, full-time employees are over-represented in this cluster. Households with higher incomes are often found in this group.

#### Cluster 2: Group with medium mental resources (39.6%)

The second group is characterized by average social support, but also above-average pessimism. With regard to dispositional optimism, this cluster hardly differs from cluster 3 (both clusters are pessimistic about the future), but receives more social support compared to the low-resource cluster. Although mental burden is slightly more pronounced compared to the total sample, mean values of these constructs are below the clinically relevant cut-off levels. General life satisfaction for this group can be described as slightly satisfied.

Of all three clusters, this group is the oldest, people over 66 years of age or retired are overrepresented. Also, widowed people are more common here. Households with below-average incomes are overrepresented. Marginal workers are more common here than in other groups. Households with two persons are more frequent in this cluster and there are often no children living in the household. First-generation migrants are also found here relatively often.

#### Cluster 3: Low-resource group (12.1%)

This small group can be described by weak resources and greater mental burden. In particular, a lack of social support, clinically relevant anxiety in a mild form and significant depressive symptoms can be identified here based on corresponding cut-off values. This group shows the lowest life satisfaction and higher than average somatic symptoms.

This group consists of considerably more men than women. Middle-aged people (46 to 65 year olds) are overrepresented. Further, people in this cluster are mostly single, divorced or widowed people, married people and people living with a partner are below average here. Accordingly, one-person households are very common in this group. Another special feature of this group is the proportion of unemployed persons who are represented here twice as often as in the overall sample. The amount of 1st and 2nd generation migrants is above average. People with below-average household income are most often found here. Compared to other groups, people from economically disadvantaged districts are more frequently represented in this cluster.

### Socioeconomic differences between clusters

In order to find out which characteristics significantly distinguish clusters from each other when testing all characteristics simultaneously, a multinomial logistic regression analysis was carried out with cluster membership as the dependent variable and sociodemographic characteristics as the independent variables. Because of its middle position, Cluster 2 was selected as the reference category: The effects of sociodemographic variables were thus compared between clusters 1 and 2 and clusters 2 and 3.

For the multinomial logistic regression analysis family status was also taken into account in the variable “living with a partner”, so that married persons living together with a spouse were assigned to the category “living with a partner”. In order to keep the number of cases evaluated as large as possible, missings were included in the analysis for the variables “origin” and “level of household income”.

Results show that only the following covariates showed a statistically significant effect: gender, number of children in the household, living with partner, occupational status, origin, level of household income. When other covariates were controlled, age no longer had a significant effect on the cluster affiliation. Living in a low-income area, which was observed more frequently in the clusters with weak mental resources in the descriptive findings, no longer showed any effect in the multivariate analysis.

The overall-test of relationship was significant at the level of *p* < 0.05. According to pseudo-*R*^2^ statistics, independent variables explain a variation in the variable cluster membership of 2.9–6.2% (Cox und Snell *R*^2^ = 0.052, Nagelkerke *R*^2^ = 0.062, McFadden *R*^2^ = 0.029). Furthermore, the Hosmer-Lemeshow test [69] was calculated for each logit equation (comparison of clusters 1 and 2, as well as clusters 2 and 3) with the respective non-significant result. The classification accuracy rate was 55.5%, whereby available predictors predicted the affiliation to cluster 1 to 71.4%, to cluster 2 to 44.9% and to cluster 3 only to 0.9%.

Results of the multinomial logistic regression analysis (see Tables 7 and 8) show that clusters 1 and 2 differ statistically significant by the characteristics gender, number of children in the household, origin, employment status and household income. Men, part-time employees, unemployed, housewives/housemen have a higher likelihood of being in cluster 2. Persons with below-average household incomes and migrants are significantly more likely to be in cluster 2, while internal migrants from West Germany and persons with children at home have a higher likelihood of entering cluster 1.

**Table 7:** Multinomial logistic regression, likelihood ratio tests, *n* = 5,940

	Model Fitting Criteria			Likelihood Quotient Tests		
	AIC of Reduced model	BIC of Reduced model	-2 Log-Likelihood of Reduced model	Chi-Square	df	Sign.
Constant term	1531.942	1746.004	1467.942	0.000	0	
How many children in household	1541.012	1741.696	1481.012	13.070	2	0.001
Gender	1541.335	1742.019	1481.335	13.393	2	0.001
Partner	1547.052	1747.736	1487.052	19.111	2	< 0.001
Origin	1533.798	1694.345	1485.798	17.856	8	0.022
Household income (categorizied)	1620.749	1808.054	1564.749	96.807	4	< 0.001
Occupational status	1559.959	1693.748	1519.959	52.017	12	< 0.001

**Table 8:** Multinomial logistic regression, parameters estimates, *n* = 5,940

Cluster number of case^a^		B	Std. Err.	Wald	df	Sign.	Exp(B)	95% C.I. for Exp(B)	
								Lower	Upper
1	Constant term	0.713	0.061	137.847	1	0.000			
	How many children in household	0.187	0.053	12.251	1	0.000	1.206	1.086	1.339
	[Gender = male]	-0.146	0.058	6.337	1	0.012	0.864	0.772	0.968
	[Gender = female]	0^b^			0				
	[partner = no]	0.119	0.148	0.643	1	0.423	1.126	0.842	1.505
	[partner = yes]	0^b^			0				
	[origin = BRD]	0.315	0.157	4.052	1	0.044	1.371	1.008	1.863
	[origin = 1st Generation migrants]	-0.472	0.202	5.472	1	0.019	0.624	0.420	0.926
	[origin = 2nd Generation migrants]	-0.104	0.081	1.658	1	0.198	0.901	0.768	1.056
	[origin = not clear]	-0.109	0.092	1.405	1	0.236	0.896	0.748	1.074
	[origin = GDR]	0^b^			0				
	[Household income = missing]	-0.173	0.116	2.208	1	0.137	0.841	0.670	1.057
	[household income < 2750 Euro /Month]	-0.604	0.068	78.964	1	0.000	0.547	0.478	0.624
	[household income > = 2750 Euro /Month]	0^b^			0				
	[Occupational status = study]	1.078	0.654	2.717	1	0.099	2.938	0.816	10.586
	[Occupational status = Houseman/housewife/maternity leave/parental leave]	-0.755	0.334	5.105	1	0.024	0.470	0.244	0.905
	[Occupational status = Unemployed]	-0.459	0.160	8.176	1	0.004	0.632	0.462	0.866
	[Occupational status = Retired]	-0.143	0.074	3.765	1	0.052	0.867	0.751	1.001
	[Occupational status = part-time/hourly employed with less than 14 h/week]	-0.282	0.183	2.374	1	0.123	0.754	0.526	1.080
	[Occupational status = working part-time with 15 to 34 h/week]	-0.258	0.100	6.670	1	0.010	0.772	0.635	0.940
	[Occupational status = Working full time (with 35 h and more/ week)]	0^b^			0				
3	Constant term	-1.804	0.120	224.453	1	0.000			
	How many children in household	0.174	0.092	3.572	1	0.059	1.190	0.994	1.426
	[Gender = male]	0.193	0.108	3.182	1	0.074	1.213	0.981	1.500
	[Gender = female]	0^b^			0				
	[partner = no]	0.896	0.201	19.933	1	0.000	2.449	1.653	3.629
	[partner = yes]	0^b^			0				
	[origin = BRD]	0.461	0.267	2.992	1	0.084	1.586	0.940	2.674
	[origin = 1st Generation migrants]	0.372	0.292	1.616	1	0.204	1.450	0.818	2.572
	[origin = 2nd Generation migrants]	-0.042	0.150	0.076	1	0.782	0.959	0.715	1.288
	[origin = not clear]	-0.046	0.174	0.070	1	0.791	0.955	0.680	1.342
	[origin = GDR]	0^b^			0				
	[Household income = missing]	0.015	0.225	0.005	1	0.946	1.015	0.653	1.578
	[household income < 2750 Euro /Month]	0.158	0.127	1.546	1	0.214	1.171	0.913	1.503
	[household income > = 2750 Euro /Month]	0^b^			0				
	[Occupational status = study]	0.606	0.931	0.423	1	0.515	1.832	0.295	11.367
	[Occupational status = Houseman/housewife/maternity leave/parental leave]	0.016	0.509	0.001	1	0.975	1.016	0.374	2.758
	[Occupational status = Unemployed]	0.561	0.208	7.316	1	0.007	1.753	1.167	2.633
	[Occupational status = Retired]	-0.384	0.141	7.413	1	0.006	0.681	0.517	0.898
	[Occupational status = part-time/hourly employed with less than 14 h/week]	-0.373	0.354	1.106	1	0.293	0.689	0.344	1.380
	[Occupational status = working part-time with 15 to 34 h/week]	-0.161	0.188	0.728	1	0.394	0.852	0.589	1.231
	[Occupational status = Working full time (with 35 h and more/ week)]	0^b^			0				

*Cluster 2* Group with medium mental resources, *Cluster 3* Low-resource group

^a^The reference category is: 2

^b^This parameter is set to zero because it is redundant

The clusters with medium (cluster 2) and low mental resources (cluster 3) were characterized by living with partner and employment status: people without a partner are more likely to be found in cluster 3. Unemployed people are more likely to be found in cluster 3 and retired in cluster 2.

Taking cluster 1 as a reference category and comparing the other groups with it (results are not presented in detail in this paper), the following factors show a significant effect in distinguishing between cluster 1 and 3: Male gender, no partner, 1st generation migrant, below-average household income and unemployment are more typical for the cluster with low mental resources.

Summarizing the results of all multinomial regressions, we conclude that being male, being unemployed, having emigrated, and having a small household income are more likely to be associated with having lower mental resources. If you also do not have a partner, you have an even higher probability of being in the particularly vulnerable group with few resources and a critical amount of mental burden. However, since membership in cluster 3 could only be explained to a small extent, we cannot really explain this group on the basis of the socioeconomic characteristics examined. In addition to the poor socioeconomic situation, this group has additional specifics, information on which is not available in this study.

## Discussion

The aim of this study was to create an empirical typology based on the population’s mental resources (social support and optimism) using a randomly-selected general population community sample representative of the German city of Leipzig.

In a two-stage process, three clusters were identified using hierarchical cluster analysis and the K-means method. Almost half of the sample was assigned to the cluster with good mental resources. Of the total sample, 12% were characterized by poor mental resources and formed the poor-resource group. Furthermore, in line with previous research, our results show that good mental resources correspond with good mental health. In the poor-resource group, almost all mental health indicators were above the clinically recognized cut-off values, indicating significant mental burden of this group. However, an uneven mix of social and psychological resources shows a slightly different picture of mental health: In addition to the group with high mental resources and the group with the lowest level of social support and optimism, we have identified a third group that has an average social support but is rather pessimistic about the future and is mentally burdened above average. In the literature, references can be found on the mediation of social support with regard to the effects of optimism on psychological stress (see e.g. [70, 71]). Differentiating between the recipients’ and providers’ perspective on social support, a study by Vollmann et al. [72] shows that optimists hold positive illusions about available support and that these illusions account at least partly for the stress buffering effect of optimism. The results of our analysis show that not only a critical amount, but already a medium endowment of mental resources is nevertheless associated with above-average psychological burden and thus poses a considerable risk to health.

In distinguishing between those with higher and lower (medium or poor) mental resources, following factors play a significant role. Male gender, unemployment, being born abroad and low household income increase the odds for having fewer mental resources. If one also does not have a partner, the probability of belonging to the particularly endangered group with critical mental resources and mental problems increases. Similar results regarding unemployment were found for ESSI in Cordes et al. [10], for F-SozU (Social Support Questionnaire) in Fydrich et al. [73] and for LOT-R in Hinz et al. [41]. Low (household) income associated with low social support occurs in the study by Cordes et al. [10] and associated with low optimism was found by Hinz et al. [41]. In contrast to our study, Cordes et al. [10] found low social support more likely to be associated with female gender. The opposite finding that men have lower social support, can be found, for example, in Hessel et al. [74] and Fydrich et al. [73]. In the studies by Hinz et al. [41], Armbruster et al. [75] and Glaesmer et al. [42] males were slightly less optimistic than females. There is also empirical evidence that men have fewer contacts with neighbors than women. Social support or networks have a stronger protective effect on mental health in women than in men [76]. Reasons why there should be differences in social support between women and men are sought in, for example, the different socialization of both genders. In socialization of women, there is usually a high value on warmth and search for intimacy, while in men, the focus is on more autonomy and independence and less expression of feelings [77].

Internal migrants from West Germany have a higher likelihood of being in the cluster with good mental resources. A nationwide survey of inner-German migrants showed that migrants who emigrated from West to East Germany were on average better educated and had higher professional positions [78]. According to the authors of the study, the male West-East migrants examined seem to have benefited from the migration in view of the lower values ​​of the mental stress. They also reported more social support than West Germans who had not emigrated. Female West-East internal migrants, on the other hand, reported more mental health problems and less social support than West German women who did not emigrate.

In our study, the number of children in the household is also a factor that increases the probability of being in the cluster with good resources. On the one hand, it may be an effect of larger households [10], in which the probability of receiving social support from a family member is higher. On the other hand, children themselves are likely to be a source of optimism.

Groups with medium and poor mental resources differ significantly only by factors that play an important role in terms of social support: living with a partner and employment. At the same time, our results show that the group with poor mental resources is very difficult to predict by socioeconomic factors. Personality factors can also play a major role here, so that, for example, extraverted people can accumulate more mental resources than introverted people in the same socioeconomic situation [79, 80].

Also interesting was the result of univariate analyzes that age groups 46 to 55 years and 56 to 65 years occur more often than average in the clusters with fewer mental resources. People in these age groups were on average 24 to 43 years old when Germany was reunified in 1990. It is reasonable to assume that people of this active working age were particularly disadvantaged by the transformations in industry and education in the course of reunification, which for many East Germans meant the end and devaluation of their professional biographies and, more often, unemployment [29, 81]. This disadvantage could lead to pessimism about the future for the people concerned. However, in the multivariate analysis, the effect of age was not significant, suggesting that remaining socioeconomic factors were more important in terms of cluster membership.

Furthermore, this study shows that clustering as a multivariate approach is well suited to identify groups in the population that are similar in terms of their mental resources. The simultaneous consideration of perceived social support and dispositional optimism in the formation of types as well as the subsequent description of the types with the indicators of psychological burden made it possible to obtain a multidimensional picture of the mental health of the studied population.

The main limitation of this study is that the sample is only representative of one large East German city. The specifics of mental resources between East and West Germany and between center and periphery could not be pursued. The participation rate in the study of 33% could also possibly cause bias, since it cannot be ruled out that those who did not take part in the study differed significantly from those who took part. Another limitation of the study relates to the measurement of the migration background of respondents. In this study, this variable was only constructed on the basis of information on the place of birth, so that it remains unknown where the respondents mostly grew up and when they emigrated. The duration of the (internal) migration is an important parameter with regard to the time that one would have to build up networks and contacts in the place of emigration. Due to the cross-sectional design of the study, no cause and effect relationships could be derived with regard to the endowment of mental resources.

## Conclusions

Results of this study indicate the importance of mental resources for mental health. Not only a weak, but even a medium mental resource endowment can go hand in hand with a considerable mental burden and thus pose a risk to mental health. The result of the study that approximately one tenth of the sample had mental resources only to a small extent, but a critical level of mental burden, deserves special attention. Characteristics such as gender, employment, origin and household income correlate with the likelihood of having fewer vs. large amounts of mental resources, where in particular male gender, unemployment, being born abroad and low household income increases the odds for having lower mental resources. This result emphasizes the need for demanding the development and implementation of low-threshold, multi-lingual, outpatient mental health care programs focusing especially of the needs of this vulnerable group in order to strengthen psychological well-being and prevent from developing further mental health problems.

## Data Availability

The availability of these data is restricted. The data are the property of the University of Leipzig (being obtained from the Leipzig Research Center for Civilisation Diseases) and are subject to the Law for the Protection of Informal Self-Determination in the Free State of Saxony (Saxon Data Protection Act). Use of data can be requested from Kristine Khachatryan (kristine.w.khachatryan@googlemail.com).
